# Phase Determination and Demonstration of Parental Mosaicism of Intragenic *PRKN* Deletions Initially Identified by Chromosomal Microarray Analysis

**DOI:** 10.3390/genes16060630

**Published:** 2025-05-24

**Authors:** Lauren A. Choate, Francis Hoffman, Jessica H. Newman, Cassandra Runke, Matthew Webley, Nicole L. Hoppman, Erik C. Thorland

**Affiliations:** 1 Division of Laboratory Genetics, Department of Laboratory Medicine and Pathology, Mayo Clinic, Rochester, MN 55905, USA; 2 Division of Cytogenetics, Center for Advanced Molecular Diagnostics, Department of Pathology, Harvard Medical School and Brigham and Women’s Hospital, Boston, MA 02115, USA

**Keywords:** *PRKN*, mosaicism, phase determination, chromosomal microarray

## Abstract

Background: Autosomal recessive juvenile Parkinson disease (ARJP) is an early-onset neurodegenerative disorder characterized by Parkinsonian motor symptoms with slow progression and preserved cognition. Biallelic pathogenic variants within the *PRKN* gene are associated with ARJP. Among *PRKN* pathogenic variants, deletions are a frequent occurrence and may be identified through chromosomal microarray testing. Methods: Here we present a case with two intragenic *PRKN* deletions initially identified as a secondary finding using chromosomal microarray. One deletion was paternally inherited and the second initially appeared to be de novo. In addition to microarray which initially identified the two deletions, long-range GAP-PCR and Sanger sequencing were used to further characterize the de novo deletion and phase of the deletions. Results: Molecular characterization of the apparently de novo deletion demonstrated low-level maternal mosaicism of this deletion, thus proving that these deletions are in trans in the proband, yielding a diagnosis of autosomal recessive juvenile Parkinson disease. Conclusions: This case highlights the utility of a diagnostic approach combining microarray, long-range PCR, and Sanger sequencing to establish the phase and confirm biallelic ***PRKN*** deletions in a patient with ARJP. Furthermore, these findings highlight the importance of investigating the possibility of parental mosaicism to determine the phase of autosomal recessive variants and establish accurate recurrence risks.

## 1. Introduction

Parkinson disease is the second-most common neurodegenerative disorder and is caused by a combination of aging, environmental factors, and genetics [1]. Both autosomal dominant and autosomal recessive mendelian forms of Parkinson disease exist, along with genetic risk loci that contribute to the formation of sporadic disease [2]. Autosomal recessive juvenile Parkinson disease (ARJP) is a form of Parkinson disease that has the classical findings of Parkinson disease, including resting tremor, muscle rigidity, and bradykinesia, but with a mean age of onset of 31 years. Progression of the disease is typically slow. Features commonly associated with progression include freezing of gait, postural deformity, and motor fluctuations; however, cognitive impairment is typically not seen [3].

ARJP has been associated with several genes; however, biallelic mutations within the *PRKN* gene (also known as *PARK2*) are the most common [4,5]. The *PRKN* gene encodes the parkin protein, which is an E3 ubiquitin ligase that targets proteins for proteasomal degradation and is thought to be involved in the maintenance of mitochondria [6,7,8]. Loss-of-function variants within *PRKN* cause disease, with both single-nucleotide variants, including missense, nonsense, and frameshift mutations, and structural variants, including deletions and duplications, being classified as pathogenic [9,10]. The loss-of-function variants result in defective or absent parkin protein, which leads to the disruption of the ubiquitin–proteasome system and the accumulation of proteins typically targeted for degradation [11].

Heterozygous *PRKN* single-nucleotide variants and structural variants may carry an increased risk of disease. Carriers of one *PRKN* mutation are more frequent among patients with Parkinson disease compared to controls [12], although they may only represent a genetic risk loci associated with the formation of sporadic disease [13]. However, the role of heterozygous carrier status in disease remains contested [14].

Structural variants are frequently found within the gene, with over 200 unique structural variants noted in population data (gnomAD SV v2.1) [15]. Greater than 0.5% of individuals within the UK Biobank cohort have copy number variants within the *PRKN* gene [14]. Several features of the *PRKN* gene contribute to the frequency of structural variants within the gene, including its localization within a common fragile site, FRA6E [16], its large size (~1.4 Mb, seventeenth-largest gene in the human genome), and the presence of intragenic repetitive elements [17].

## 2. Materials and Methods

### 2.1. Chromosomal Microarray

Chromosomal microarray (CMA) analysis was performed using the Applied Biosystems (Affymetrix, Santa Clara, CA, USA) CytoScan HD platform, which has 1.9 million copy number probes and 750,000 single-nucleotide polymorphism (SNP) probes. Peripheral blood specimens from the proband, mother, and father were processed according to the manufacturer’s instructions. Data were analyzed using Chromosome Analysis Suite (ChAS) software version 3.3 (Thermo Fisher, Waltham, MA, USA) and reported using the NCBI human genome build 37.1 (hg19). The genotypes on chromosome 6 were extracted from each specimen using the ChAS software. The phase of the deletion was determined by comparing the proband’s genotypes within and surrounding deleted interval in the *PRKN* gene with the genotypes from the parents. Out of the 79 SNPs in the interval, 14 were informative for phase determination. The data presented reflect clinical testing performed within our diagnostic laboratory.

### 2.2. Long-Range PCR and Sanger Sequencing

GAP PCR and Sanger sequencing were performed on DNA extracted from the proband, maternal sample, and an unrelated control sample. PCR primers were designed to flank the edges of the deletion and anneal outside the area where the proband had informative SNPs for paternal inheritance. Primer sequences were TCCCATCACACCAGAAAACA and CTTGGGAGAAGGCAGAATGA. Long-range PCR was performed using the TaKaRa LATaq Hot Start Version according to the manufacturer’s instructions. The long-range PCR product was amplified with the following ~10 h thermocycling program:

1. Hold: 94 °C for 1 min
2. Denature: 95 °C for 15 min
3. Anneal: 65 °C for 30 min
4. Extend: 68 °C for 15 min
5. Steps 2–4, 35 cycles
6. Final extension: 72 °C for 10 min

The long-range PCR product was size-confirmed with a gel and purified using the AMPure XP purification kit. The purified product was Sanger-sequenced using the BigDye Terminator v1.1 Cycle Sequencing kit with UPS universal primer sequences GGGTTCCCTAAGGGTTGGA and GTGCCAGCAAGATCCAATCTAGA. Fastq files from Sanger sequencing were aligned to hg19 using BLAT to clarify the breakpoints in the proband and mother’s samples.

## 3. Results

**Figure 1:**
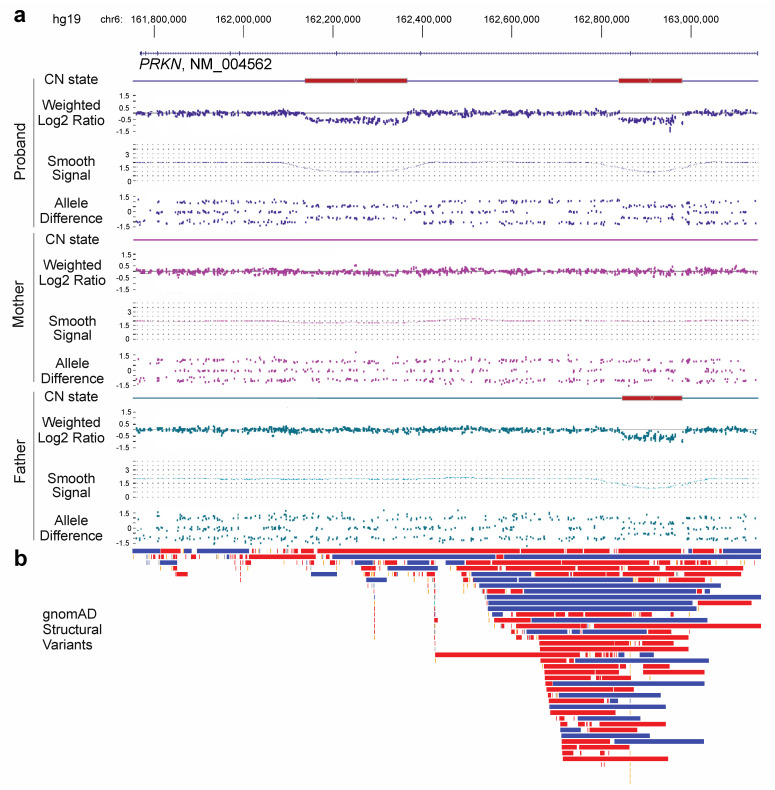
*PRKN* deletions. (**a**) Chromosomal microarray data for the proband, mother, and father at the *PRKN* gene. Two deletions are found in the proband. The exon 2 deletion is paternally inherited, while the exon 7 deletion appears to be de novo. (**b**) Structural variants (deletions in red, duplications in blue, and inversions in orange) found in a normal population cohort (gnomAD SV v2.1).

Chromosomal microarray (CMA) testing was ordered for a peripheral blood specimen from a six-day old female with a family history of a 1.5 Mb 17q12 duplication. This recurrent duplication exhibits incomplete penetrance with 90% of cases being inherited. Reported phenotypes of the duplication include variable intellectual disability, speech and motor delay, hypotonia, and seizures [18]. The familial 1.5 Mb duplication was observed on chromosome 17 and included 41 genes. In addition to this duplication, two non-overlapping intragenic deletions were found within the *PRKN* gene on 6q26 (Figure 1a). These deletions were approximately 140 Kb and 227 Kb in size and encompassed exons 2 and 7 (NM_004562.2), respectively, based on CMA. Both deletions were predicted to be out-of-frame and resulted in loss of *PRKN* gene function; however, it was unclear if these deletions were in cis or in trans.

Parental CMA studies were performed to assist in phase determination for the proband’s *PRKN* deletions. The exon 2 deletion was clearly paternally inherited; however, the exon 7 deletion appeared to be de novo. Informative single-nucleotide polymorphisms (SNPs) from the CMA genotype data within and surrounding the exon 7 deletion supported paternal inheritance of the intact copy, suggesting that the deletion arose from the maternal homolog (Table 1). Interestingly, closer inspection of the maternal copy number probes from CMA demonstrated possible low-level mosaicism for the deletion below the limit of detection for the Cytoscan CMA platform.

**Figure 2:**
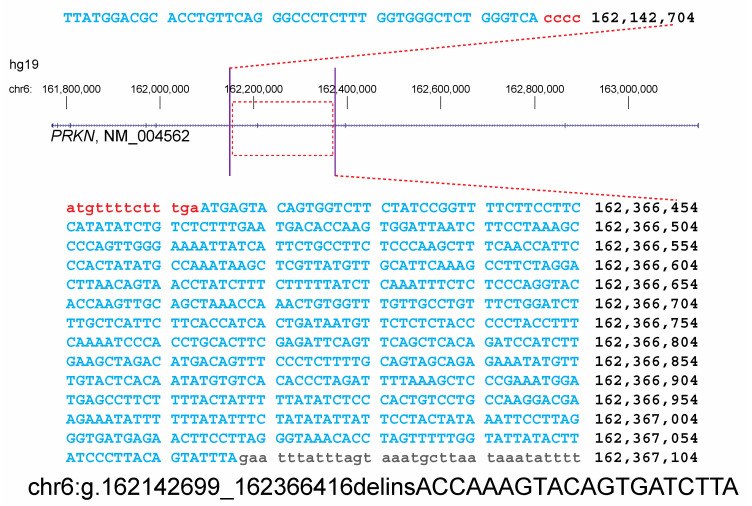
Breakpoint detection of the maternally inherited deletion. Sanger sequencing of the long-range PCR product demonstrates a 223.7 kb deletion with breakpoints at chr6: 162,142,699 and 162,366,416, as well as a 20 bp insertion, with the following nomenclature: chr6(GRCh37):g.162142699_162366416delinsACCAAAGTACAGTGATCTTA. Bases in blue align to the genome and bases in red are absent in the Sanger sequencing product.

Primers were designed flanking the exon 7 deletion to perform long-range GAP PCR in the proband, mother, and a control. In the absence of the deletion, the product should be too large to amplify given the thermocycling conditions. No product was identified in the control sample; however, a PCR product was produced in both the proband and mother. Sanger sequencing of this long-range PCR product confirmed that both the proband and mother carry the deletion. The size of the deletion was refined as 223.7 kb, with a breakpoint nomenclature of chr6(GRCh37):g.162142699_162366416delinsACCAAAGTACAGTGATCTTA (Figure 2). These data confirmed that the proband’s deletions are in *trans*. Thus, the proband has a molecular diagnosis of ARJP.

## 4. Conclusions

We provided a diagnosis of ARJP in a newborn child based on a secondary finding of two intragenic deletions within the *PRKN* gene identified through CMA. It is unclear when or how the disease will manifest in this patient, as age of onset, progression, and clinical symptoms can vary greatly [19,20,21,22]. Modifier genes may also contribute to the disease state [23]. The confirmation of carrier status for each parent changes the reproductive risk for this couple.

Due to the structure of the *PRKN* gene, deletions are thought to be recurrent, independent events while point mutations may be attributed to founder effects [24]. Accordingly, the deletions identified in the proband are localized to regions where structural variation is present in a control population, particularly the deletion containing exon 2 of *PRKN*; however, these deletions have not been reported before (Figure 1b). Heterozygous copy number variation within *PRKN* is a relatively common finding in patients that have received CMA testing in our laboratory, and the reporting of heterozygous copy number changes in this gene should be carefully considered.

We used a combination of methods to confirm that the proband in this case had biallelic deletions in the *PRKN* gene by identifying low-level maternal mosaicism. Methods such as CMA, multiplex ligation-dependent probe amplification (MLPA) [25], digital-droplet PCR (ddPCR) [26], and optical genome mapping (OGM) [27] have all been used to identify deletions and duplications in *PRKN*. Because of the large size of the *PRKN* gene, GAP-PCR was used for phase determination of the proband in this study due to the suspected maternal mosaicism. However, other methods may be utilized when the mode of inheritance is not clear or is de novo. Custom FISH probes were used to determine the phase of a compound heterozygote with de novo and inherited deletions [28]. RT-PCR has also been used for phase determination in a cohort study [29], which is simpler to design if the correct specimen is available. Trio whole genome sequencing is another option for phase determination as the underlying informative genotype data should resolve the parent haplotype of origin, as was the case in the study presented here at a smaller scale. In addition, long-read sequencing has been used to resolve a complex inversion involving the *PRKN* gene and could be used for phase determination [30]. As methods in cytogenomics evolve, there may be improved ways to determine phase of structural rearrangements, particularly in large genes such as *PRKN*. For now, a comprehensive approach using confirmatory molecular methods is the best method to ensure proper phasing.

When multiple variants are detected in genes associated with autosomal recessive disorders, it is essential to determine the phase to differentiate between a diagnosis and carrier status and to determine recurrence risks. Frequently, parental studies are sufficient to make this determination if neither variant is de novo. However, this case demonstrates the importance of additional follow-up studies when apparent de novo variants are detected. Low-level parental mosaicism should be considered and tested for using alternative methods, especially in the case of autosomal recessive disorders, and has significant implications for recurrence risk.

## Figures and Tables

**Table 1:** Informative SNPs. Informative single nucleotide variants within the deleted interval containing exon 7 show paternal inheritance (bolded alleles are shared between father and proband).

SNP	Position	Mother	Proband	Father
rs6907465	162250726	TT	**AA**	**A**T
rs9347543	162252602	AA	**GG**	**G**A
rs9458393	162255535	GG	**TT**	**T**G
rs6926642	162270981	TT	**CC**	**C**T
rs9365329	162271943	TT	**GG**	**G**T
rs9347547	162280309	GG	**AA**	**A**G
rs7750426	162281372	TT	**GG**	**G**T
rs2186803	162281492	AA	**CC**	**C**A
rs2155486	162281533	TT	**AA**	**A**T
rs6935164	162282924	CC	**TT**	**T**C
rs9689946	162283949	GG	**TT**	**T**G
rs9355958	162322332	TT	**CC**	**C**C

## Data Availability

The original contributions presented in the study are included in the article; further inquiries can be directed to the corresponding author.
